# Sexual Knowledge among High School Students in Northwestern Iran

**DOI:** 10.5402/2012/645103

**Published:** 2012-08-27

**Authors:** Ayyoub Malek, Ali Reza Shafiee-Kandjani, Abdolrasool Safaiyan, Hamid Abbasi-Shokoohi

**Affiliations:** ^1^Department of Psychiatry, Clinical Psychiatry Research Center, Tabriz University of Medical Sciences, Tabriz, Iran; ^2^Clinical Psychiatry Research Center, Tabriz University of Medical Sciences, Tabriz, Iran; ^3^Department of Psychiatry, Razi Mental Hospital, El-Goli Boulevard, P.O. Box 5456, Tabriz, Iran; ^4^Department of Vital Statistics and Epidemiology, Faculty of Health and Nutrition, Tabriz University of Medical Sciences, Tabriz, Iran; ^5^Department of Social Sciences, Tabriz University, Tabriz, Iran

## Abstract

*Objectives*. Regarding the importance of sexual desire in adolescence period and public and parental concerns about it and considering the lack of studies on sexual knowledge especially in adolescents in Iran, this study was conducted to evaluate the sexual issues in high school girls and boys. *Patients and Methods*. The cross-sectional study was performed on 2700 high school students. Students were selected through a multi-staged randomized sampling method in Tabriz and by the convenience method in Ardabil and Urmia. Data collection tool was a questionnaire including knowledge questions and measured by a three-point scale. Data were statistically analyzed with SPSS version 11.5 software. *Results*. 11.8% of the students had a low sexual knowledge, 46.7% had average and 41.5% had a high one. There was no significant difference between male and female students' general sexual knowledge. The highest knowledge was about being aware of the religious rules concerning sex. *Conclusion*. Adolescents should be trained and acquire information about the different aspects of sexual issues. Female adolescents especially need to be educated about puberty physiology, fertility physiology and STDs.

## 1. Introduction

Sexual drive is a part of natural development initiating with sexual curiosity in childhood followed by a sudden interest in exploring his/her sexual organs and peers' bodies in adolescence as well. During this period, there may be societal and parental concerns in which lack of sufficient education about adolescents' sexual issues may be mentioned. 

In religious communities like Iran, there are specific sexual norms and morality. Therefore, it seems that Muslim parents or individuals and the media, which communicate with teenagers, cannot pose sexual issues among them conveniently or they may feel uncomfortable to give sexual training to their children and teenagers. 

Transcultural studies show that human sexual behavior, in addition to biological factors, is strongly affected by culture [1].

In a study among African-American adolescents with a mean age of 15.3 years, 70 percent were reported to be sexually active and the mean age of first sexual intercourse was 11.7 years. Also, it was demonstrated that girls compared with boys had more information about AIDS, less sexual partners, more positive attitude about careful sexual behavior, and assessed themselves with higher control in comparison with boys [2].

Ina study among adolescent males in Tehran, Iran, 28 percent of the sample reported having engaged in sexual activity. Sexual experience was associated with older age, access to satellite television, alcohol consumption, and permissive attitudes toward sex. Substantial proportions of respondents held misconceptions regarding condoms, STDs, and reproductive physiology. Attitudes toward premarital sex were more permissive among respondents who were older, were not in school, had work experience, had access to the Internet or satellite television, lived separately from their parents, or reported having used alcohol, cigarettes, or drugs [3]. 

Another study showed that the knowledge level of female high school girls in two high schools in northern and southern Tehran were similar and in medium level (52.3%). According to the findings, the knowledge level of students about AIDS was insufficient and performing more studies to evaluate the role of educational programs in obtaining higher information about AIDS and its prevention was recommended [4].

Results of a study showed that knowledge level of girls about puberty physiology and complete description of somatic, psychiatric, and social puberty was poor and consequently they would experience severe fear and concern and psychological distress about abnormal feature of menstrual cycle at initiation [5].

In a study about the effect of cooperation between public sector, university, and health sectors for improving the adolescents' health education, data analysis before educational intervention showed that girls had good knowledge and attitude about marriage and pregnancy-related issues. However, their knowledge and attitude was poor about adolescents' health and puberty [6].

In a study among the couples attending to marriage counseling centers in northern and northeastern Tehran in 2006 toward correct sexual relationships, the authors concluded that young couples had poor knowledge about appropriate sexual relationships [7].

Totally, in review of studies about the sexual knowledge among students in Iran, there was no multidimensional study performed or published about different aspects of sexual knowledge in male and female students. However, in a limited manner and in special groups, some aspects of sexual matters were evaluated. Generally, regarding the importance of sexual desire in adolescence period and public and parental concerns about it and considering the lack of studies about sexual knowledge especially in adolescents in Iran, this study was conducted to evaluate the sexual issues in high school girls and boys in three large centers in provinces in northwestern Iran including Tabriz, Urmia, and Ardabil cities to respond to the following questions.

How much and what type of sexual knowledge are present in the understudy population?

Is there any difference in sexual knowledge regarding to the sex and educational level?

## 2. Patients and Methods 

This descriptive cross-sectional study was performed to evaluate the rate and type of sexual knowledge among students. The ethical committee of Tabriz University of Medical Sciences approved our study. The target population included all of the high school students in Tabriz, Urmia, and Ardabil (three major and central cities in northwestern Iran) that were collectively 125,377 subjects according to available data in education and training offices in these cities.

According to Cochran sampling formula, the sample size was calculated to be 1341 students for Tabriz, 726 for Urmia, and 633 for Ardabil. So, totally 2700 subjects were enrolled.

According to the education and training offices in Tabriz, Urmia, and Ardabil cities, there were five, three, and three districts, respectively; so six schools for each district in Tabriz (totally 30 schools), three schools for each district in Urmia (totally nine schools), and three schools for each district in Ardabil (totally nine schools) were selected.

The schools were selected based on a randomized sampling method in each city. Then the cases were selected by stratified random sampling method in Tabriz, but by Quota sampling method in Urmia and Ardabil (i.e., convenience method for selecting students with regard to gender and educational grade proportion). 

Data collection tool was a questionnaire including knowledge questions and measured by three-point scale including true, false, and no knowledge. The questions about sexual knowledge were in five domains containing 30 items: puberty physiology (5 items), fertility physiology (12 items), sexually transmitted diseases (STDs) (6 items), contraceptive methods (3 items), and religious rules regarding sexual issues (4 items). For each one of these items three options were suggested including true, false, and no information to be chosen by the students according to their knowledge. Each one of these options were allocated two, one, and zero points, respectively, and finally total score for sexual knowledge was calculated. In the total score calculation, two procedures were performed; first the false designed sentences that in them the false option should be selected had two points, and second, according to the reviewers' comments participating in questionnaire designing, five items with higher importance were weighted and their scores were two-fold multiplied. Accordingly, if a student responded correctly to all questions, the total score was 70. After the total score was calculated, the students were divided into three categories including those with low, medium, and high sexual knowledge. The total scores of zero to 20 were considered low, 21 to 40 medium, and 41 to 70 was considered high level.

The face and content validities of questionnaire were determined according to comments of psychiatrists, psychologists, sociologists, obstetricians, and clergymen. Using the splitting method, the reliability of the questionnaire was assessed by fulfilling the questionnaire by 300 students educating in first to third grades of high school. The Cronbach's alpha coefficient for first part was 0.6455 and for the second part 0.6295.

The obtained data were analyzed by SPSS statistical software using descriptive (frequency, and percent, mean, and standard error of mean) and analytical (chi-square, Fisher's exact test and *t* test) statistics.

## 3. Results

### 3.1. Total Sexual Knowledge in Students

The mean ± SE of total sexual knowledge was 35.95 ± 0.248. According to Table 1, the majority of students had medium level of sexual knowledge. According to chi-square test, difference between different levels of sexual knowledge was significant.

### 3.2. Sexual Knowledge according to the Gender

As shown in Table 2, there was significant difference between sexual knowledge among girls and boys, as boys' sexual knowledge was higher than girls (*P* = 0.001). 

### 3.3. Sexual Knowledge according to the Educational Grade

As shown in Table 3, the sexual knowledge in majority of students was in medium level in all three educational grades, as among which the third grade students had the highest percentage of sexual knowledge.

### 3.4. Sexual Knowledge according to the Type of the Knowledge

As shown in Table 4, among the different sexual knowledge domains, the highest level of sexual knowledge was about religious rules including major ritual ablution (Ghosl), permitted sexual relationships, masturbation, and adultery. The lowest level of sexual knowledge was about contraceptive methods and STDs.

### 3.5. Sexual Knowledge in Different Types according to the Gender

As shown in Table 5, all different types of sexual knowledge significantly differed among girls and boys. So that, the boys had higher means in regard to sexual knowledge about puberty physiology, fertility physiology, and STDs, while girls were higher in sexual knowledge of contraceptive methods and religious rules. 38% of boys had high level of knowledge about puberty physiology while this was only 23% in girls. Also 48 percent of girls had low knowledge about STDs, and this was 32 percent in boys. On the other hand, 20 percent of boys and three percent of girls had high knowledge about STDs.

## 4. Discussion

The results of current study showed that the majority of students had medium level of sexual knowledge, and only 27 percent had high sexual knowledge. The mean sexual knowledge score was different between girls and boys. When different types of sexual knowledge were evaluated, the awareness was highest for the religious rules and lowest about the contraceptive methods and STDs (Table 4). Also, the knowledge about the different types of knowledge was different between girls and boys, the boys achieved significantly higher mean scores in puberty physiology, fertility physiology, and STDs.

Unfortunately, there is no comprehensive and multidimensional study performed or published in Iran about different aspects of sexual knowledge in students; but in a limited form and in special groups some aspects of sexual issues have been evaluated. The results of these studies are in congruence with the findings in our study that showed the sexual knowledge is high in only 27 percent and is mainly in low and medium levels; Gachkar and Amini reported a low knowledge level of the AIDS among female high school girls in Tehran and performing more studies to evaluate the role of educational programs in obtaining higher information about AIDS prevention was recommended by them [4]. Results of a study by Ahmadi and Malekafzali about health educational needs of adolescent third intermediate school girls in south of Tehran showed that knowledge level of the girls about puberty physiology and complete conception of biopsychosocial aspects of puberty was poor [5]. In a preliminary study by Malek Afzali, the improvement of knowledge and attitude of fertility and menstrual heath in 12–14 year-old adolescent girls in a Heath Care Center, Semnan, Iran, were evaluated and reported that the knowledge, attitude, and behavior of these girls are poor or inappropriate about adolescents health and puberty [6]. A study showed that the knowledge level of the girls about the contraceptive methods, STDs, AIDS, and sexual health is insufficient [8]. 

The important point in the above-mentioned and the other similar studies in Iran is that those are generally performed among girls and all reported a low awareness about the sexual issues. In our study, after comparison of the mean scores of different types of sexual knowledge, the girls achieved significantly lower scores in puberty physiology, fertility physiology, and STDs subscales, compared with boys.

The obtained results show that the students generally do not acquire the necessary knowledge and information about the sexual issues by appropriate educational programs and also demonstrate the necessity for educating these issues. This question here may be arisen if educating these issues would result in more interest in sexual behaviors among adolescents. In a systematic review by World Health Organization, 52 studies were evaluated. Among 47 studies that evaluated the performed interventions, 25 studies were reported that giving information about AIDS and sexual issues resulted in no increased or decreased rate of sexual behaviors, unwanted pregnancies, and STDs. Seventeen studies reported a delay in the initiation time of sexual relationships, and a decrease in sexual partners' number, unwanted pregnancies, and STDs frequency. Only three studies reported an increased sexual behavior that in those studies there were methodological problems in designing the study and analysis of the data. Finally the authors concluded that sexual issues training would not result in increased interest to sexual behaviors. If any effect is observed it is to delay the initiation of sexual relationships and effective use of contraceptives [9].

Some studies in Iran have evaluated the view of key subjects about the training of sexual issues. In a study for determination of attitude of key public subjects including parents, clergymen, and school trainers about the education of sexual issues to adolescents, 896 subjects were evaluated. The results showed that 93 percent of parents, 86 percent of clergies, and 81 percent of school trainers were congruent with training sexual issues. However, 95 percent of parents and 55 percent of school trainers had never educated the children and students the sexual issues and 14.3 of clergymen participated in training of sexual issues to adolescents in a range of minimal to maximal. Most of the evaluated subjects accepted the need for training both sexes and the parents mentioned the best time for education to be the first stages of marriage and by health-promotion centers. The clergies and school trainers believed that training should be initially performed by parents and in first puberty stages [10]. In a study for evaluation of attitude of 400 teachers in Kerman, Iran, about the sexual education and evaluation of their view about the necessary points to be trained, it was demonstrated that the teachers achieved 65.7 percent of total attitude score about sexual training to adolescents. About the necessary sexual points to be trained, 83 percent of teachers agreed that normal menstrual cycle and related mechanisms and religious rules about it, and different aspects of AIDS (including causes, prevention, and transmission) should be completely trained to the adolescents. However, 26 percent completely disagreed with education about abortion in girls, six percent did not agree with training about sexual function of opposite sex, and 22 percent completely disagreed with training about contraceptive methods [11].

In another study, 96 percent of teachers agreed with prepubertal training of sexual issues to the girls and 91 percent out of these subjects mentioned that the educational curriculum should include introduction with puberty and menstruation, puberty-related physical and psychological changes, fears and concerns in this period, preventive methods for problems, and also introduction with socially appropriate moral issues and behaviors and religious instructions [5].

The results of a study by Noohi et al. showed that the postmarital training solely cannot be effective in information acquiring about this field and developing premarital training especially in public training centers such as high schools and universities would prepare good help for improvement of sexual relationships and having higher mental health and result in better emotional conditions for young couples and lower marital discordances and divorce frequency in the public [12]. Also, according to a meta-analysis from 1960 to 1977, the positive effect of sexual training programs on sexual knowledge is approved [13].

Totally, the results of the above-mentioned studies show that in understudy populations there is a positive attitude about the sexual training to adolescents among parents, religious directors, teachers, and school trainers, but there are some controversies about the educational content, time of education, and trainer subjects and centers. Also, regarding to sociocultural conditions in our society and content of sexual issues, this essential question proposed that if the training about the sexual issues is necessary, the most appropriate method for training is which. This is the question that needs further evaluation by researchers.

According to the results of present study, the adolescents should be trained and acquire information about the different aspects of sexual issues. Female adolescents especially need to be educated about puberty physiology, fertility physiology, and STDs. To do this, the schools, especially high schools, need a training program in different educational grades according to the cognitive, emotional, and social development of students during a curriculum and by teaching books or lessons in different educational grades.

The main limitation of this study was using nonrandom sampling method for selection of the students in two cities out of three (i.e., Urmia and Ardabil cities). However the selection of schools in these cities was in a random manner and there was a quota sampling method for each educational grade and gender. Non-random sampling method among the students may lead to a decreased generalizability of the results. But the large sample size is a positive point in our study that may moderate the effects of non-random sampling in the above-mentioned cities. 

## Figures and Tables

**Table 1:** Frequency and percent of total sexual knowledge in students.

Sexual knowledge	Low	Medium	High	*P* value
Frequency (%)	486 (18)	1485 (55)	729 (27)	0.000

**Table 2:** The mean and standard error of sexual knowledge in girls and boys.

Sex	Sexual knowledge		
	Mean	SE	*P* value
Boys	36.7	0.361	0.001
Girls	35.2	0.339	

**Table 3:** The percent of sexual knowledge in different educational grades.

Grade	Sexual knowledge			
	Low	Medium	High	*P* value
First	211 (18%)	609 (52%)	351 (30%)	0.000
Second	84 (11%)	414 (54%)	268 (35%)	
Third	76 (10%)	473 (62%)	214 (28%)	

**Table 4:** The percent, mean, and standard error of sexual knowledge in different types of knowledge.

Type of knowledge	Sexual knowledge					
	Low	Medium	High	Mean	SE	*P* value
Religious rules regarding sexual issues	513 (19%)	810 (30%)	1377 (51%)	2.25	0.043	0.000
Fertility physiology	387 (14%)	1431 (53%)	882 (33%)	2.1	0.041	
Puberty physiology	486 (18%)	1593 (59%)	621 (33%)	2.05	0.039	
STDs	1323 (49%)	1350 (50%)	27 (1%)	1.35	0.026	
Contraceptive methods	1512 (56%)	918 (34%)	270 (10%)	1.3	0.019	

**Table 5:** The mean and standard error of different types of knowledge in girls and boys.

Type of knowledge	Sexual knowledge		
	Mean	SE	*P* value
Puberty physiology			
Boys	6.35	0.063	0.000
Girls	5.10	0.059	
Fertility physiology			
Boys	17.5	0.152	0.000
Girls	16.3	0.161	
STDs			
Boys	6.7	0.182	0.000
Girls	4.9	0.083	
Contraceptive methods			
Boys	2.2	0.039	0.000
Girls	3	0.051	
Religious rules			
Boys	7.9	0.079	0.000
Girls	8.4	0.090	
